# Gut microbiota and its metabolites with thyroid diseases: functions and mechanisms

**DOI:** 10.3389/fimmu.2026.1818380

**Published:** 2026-05-21

**Authors:** Chao Jia, Min Li, Xianqiang Yu

**Affiliations:** 1 Department of Critical Care Medicine, Qingdao Municipal Hospital (Qingdao Hospital of Rehabilitation University), Qingdao, China; 2 Department of Thyroid Disease Diagnosis and Treatment, Qingdao Municipal Hospital (Qingdao Hospital of Rehabilitation University), Qingdao, China

**Keywords:** gut microbiota, gut-thyroid axis, Hashimoto’s thyroiditis, short-chain fatty acids (SCFAs), thyroid cancer, thyroid diseases

## Abstract

The gut microbiota, a complex and dynamic ecosystem of microorganisms residing in the gastrointestinal tract, has emerged as a critical regulator of human physiology, extending its influence far beyond digestion. Recent advances have illuminated the existence of a bidirectional communication network known as the “gut-thyroid axis” which posits that the intestinal microbial community and its vast repertoire of bioactive metabolites are fundamental modulators of thyroid health and disease pathogenesis. This comprehensive academic review synthesizes current evidence to elucidate the multifaceted functions and mechanisms through which gut microbiota and its metabolites, such as short-chain fatty acids, secondary bile acids, and lipopolysaccharides, impact thyroid disorders. We detail how these microbial components regulate systemic immunity, influence thyroid hormone metabolism, maintain intestinal barrier integrity, and modulate nutrient absorption, thereby contributing to the development and progression of autoimmune thyroid diseases, thyroid dysfunction, and potentially thyroid cancer. Furthermore, the review discusses the translational implications of this knowledge, including the potential for microbiota-targeted interventions like probiotics, prebiotics, and dietary modifications as adjunctive strategies in thyroid disease management. By integrating findings from human studies and animal models, this review aims to provide a mechanistic framework for understanding the gut-thyroid connection and to highlight promising avenues for future research and precision medicine approaches in endocrinology.

## 1 Introduction

Thyroid diseases, particularly autoimmune conditions such as Hashimoto’s thyroiditis and Graves’ disease, represent a significant global health burden with a rising incidence (1–8). The pathogenesis of these disorders is multifaceted, involving a complex interplay of genetic predisposition, environmental triggers, and immune dysregulation (9–14). For decades, research has primarily focused on these intrinsic host factors. However, a paradigm shift is underway with the emergence of the gut-thyroid axis, a conceptual framework that positions the gut microbiota as a critical extrinsic regulator of thyroid physiology and pathology (15–19). This axis suggests that distant communication between the gut and the thyroid is not merely coincidental but is a fundamental determinant of health and disease.

**Figure 1:**
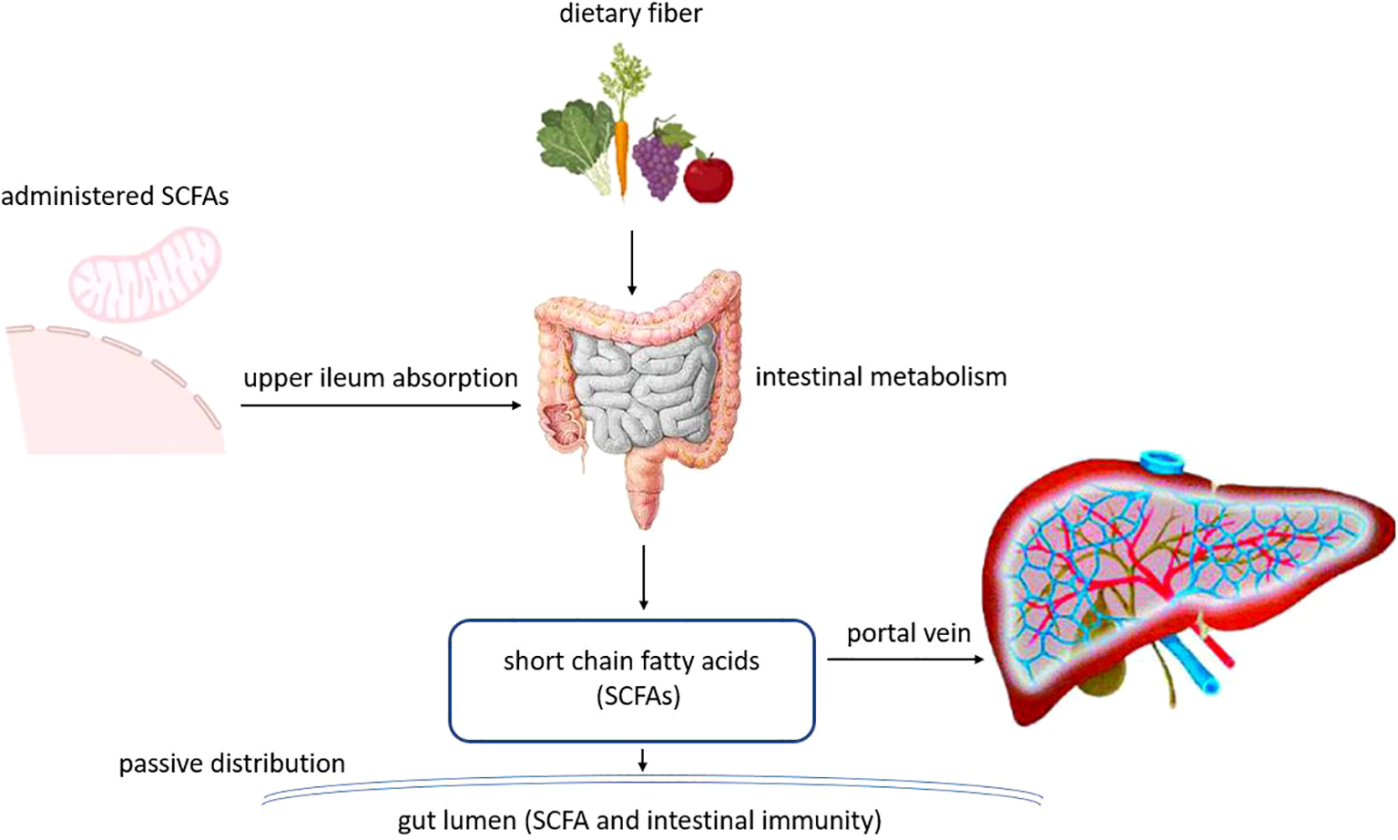
The metabolic process of SCFAs in the intestine.

The human gut microbiota, often referred to as a “microbial organ,” comprises trillions of bacteria, viruses, fungi, and archaea that co-evolve with the host. It performs indispensable functions, including nutrient metabolism, defense against pathogens, and, most crucially, the education and modulation of the systemic immune system. Beyond these roles, the microbiota produces a myriad of bioactive metabolites such as short-chain fatty acids (SCFAs), secondary bile acids, and indole derivatives which act as key signaling molecules (20–24). These metabolites can enter systemic circulation, directly or indirectly influencing host metabolism, immune responses, and endocrine signaling (**Figure 1**). This positions the gut and its microbial inhabitants not as a passive digestive unit but as an active endocrine and immunomodulatory interface with far-reaching systemic effects, capable of influencing organs far beyond the gastrointestinal tract, including the thyroid gland.

**Figure 2:**
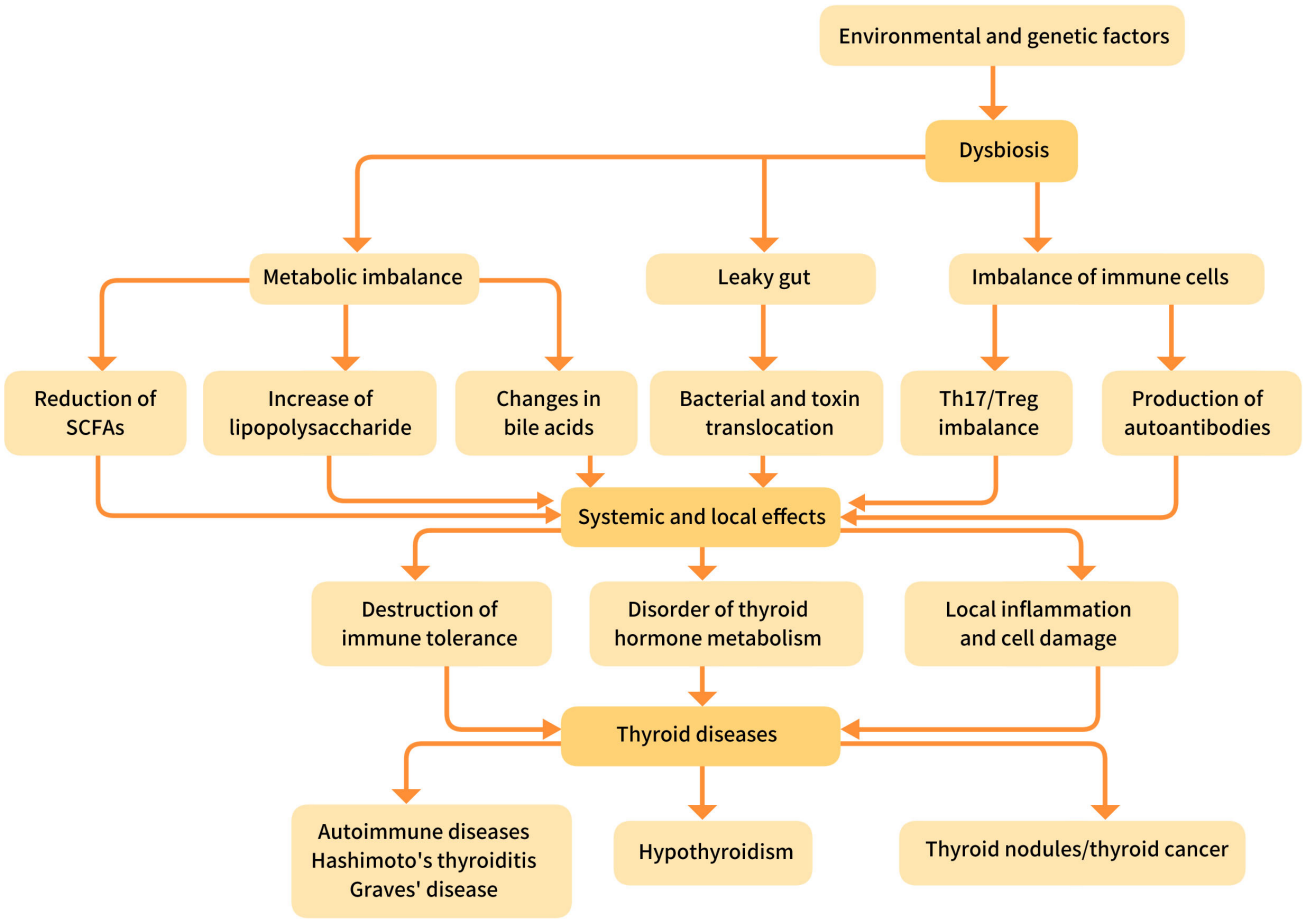
The relationship between gut microbiota and thyroid diseases.

This academic review aims to synthesize current evidence and provide a comprehensive analysis of the intricate relationship between the gut microbiota, its metabolic output, and thyroid diseases (**Figure 2**). We will delve into the core mechanisms—immune modulation, endocrine disruption, maintenance of intestinal barrier integrity, and nutrient regulation—through which the microbiota exerts its influence. By examining the altered microbial signatures and metabolic profiles associated with specific thyroid disorders, we will explore how dysbiosis can initiate and perpetuate disease. This review systematically focuses on the functional mechanisms by which specific microbial metabolites directly regulate thyroid hormone synthesis, peripheral conversion, and immune homeostasis. Furthermore, we critically examine the underexplored bidirectional interactions between gut microbiota and common thyroid medications, highlighting how microbial activity may influence drug efficacy and metabolic outcomes. Collectively, this review provides a mechanistic and translational framework that moves beyond correlation toward causation, offering new insights for microbiota-based diagnosis and targeted therapy in thyroid disorders. Ultimately, this review seeks to elucidate the functional and mechanistic basis of the gut-thyroid axis, evaluate its translational potential for novel diagnostic and therapeutic strategies, and highlight critical gaps in knowledge to guide future research in this rapidly evolving field.

### 1.1 The conceptual foundation of the gut-thyroid axis

**Figure 3:**
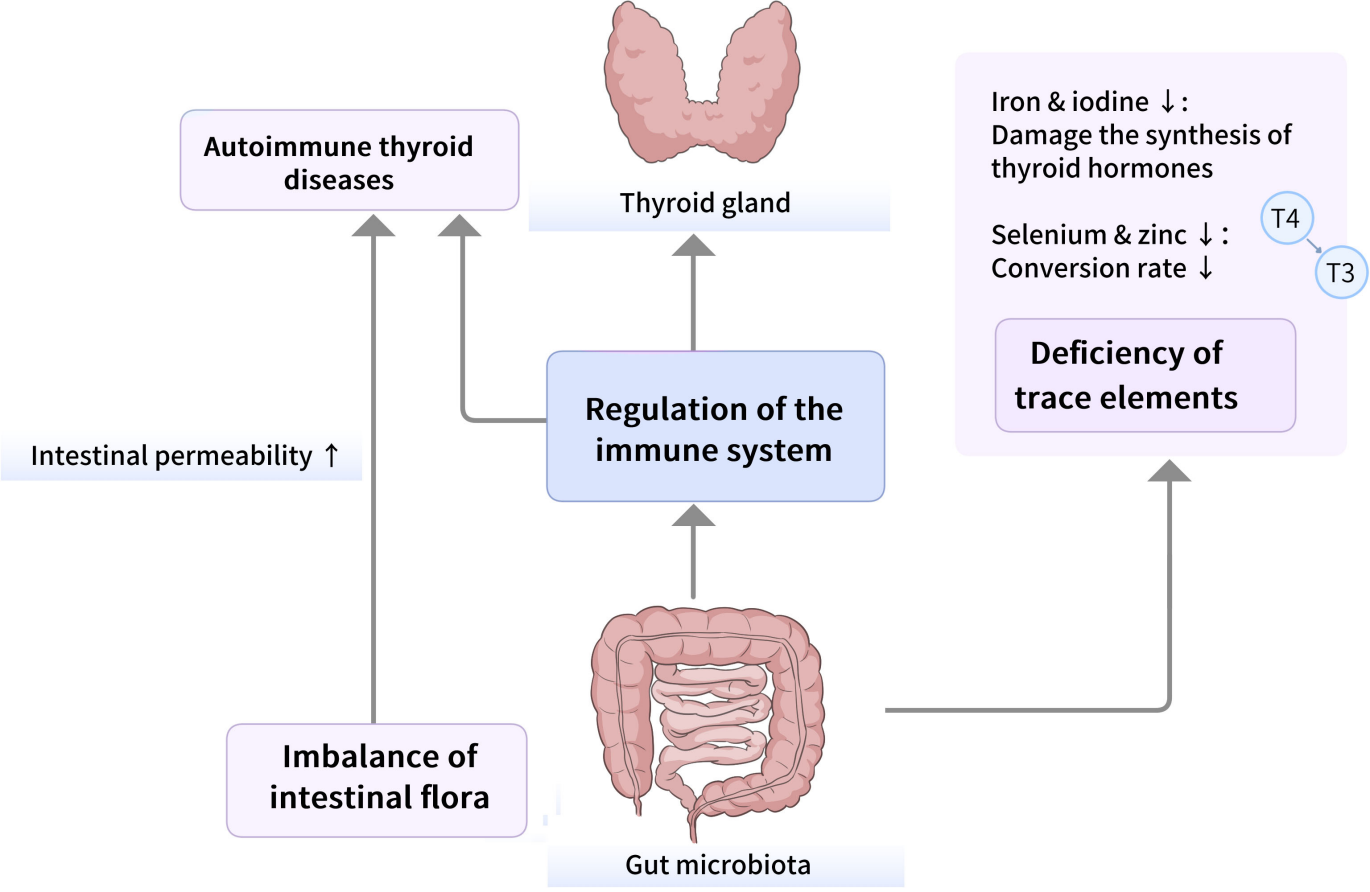
Schematic diagram of the gut-thyroid axis.

The conceptualization of the gut-thyroid axis represents a pivotal evolution in endocrinology, shifting the understanding of thyroid health from an isolated glandular system to one deeply integrated with the gastrointestinal ecosystem. This framework posits the existence of a dynamic, bidirectional communication network between the gut microbiota—the trillions of microorganisms residing in the human intestine—and the thyroid gland (**Figure 3**). The axis is built upon the fundamental recognition that the gut microbiota functions as a virtual endocrine organ, capable of producing and modulating a vast array of bioactive molecules that influence distant organs (25–27). For the thyroid, this means its function is not solely governed by the classic hypothalamic-pituitary-thyroid (HPT) axis but is also profoundly shaped by signals originating from the gut (28–30). This paradigm challenges traditional views by introducing intestinal microbial composition and metabolic output as critical, modifiable environmental factors in thyroid physiology and the pathogenesis of its diseases, from autoimmune disorders to functional impairments.

The establishment of this axis is substantiated by converging evidence from multiple scientific disciplines. Clinically, robust associations are consistently observed between altered gut microbial communities (dysbiosis) and various thyroid disorders, including Hashimoto’s thyroiditis (HT), Graves’ disease, and hypothyroidism. In the context of thyroid diseases, dysbiosis can disrupt the production and bioavailability of key microbial metabolites, impair the absorption of essential micronutrients, and alter local and systemic immune responses, thereby contributing to the pathogenesis of autoimmune thyroid disorders and affecting thyroid hormone metabolism. Experimentally, animal models demonstrate that manipulating the gut microbiota through probiotics, antibiotics, or fecal microbiota transplantation can directly alter thyroid hormone levels, modulate autoimmune responses against thyroid tissue, and change the course of thyroid disease (31–35). A pivotal experimental study provided direct causal evidence by showing that transplanting gut microbiota from Graves’ disease (GD) patients into mice successfully induced a GD phenotype, including elevated serum FT4 and TRAb levels and decreased TSH, confirming that gut microbiota dysbiosis plays a crucial role in GD pathogenesis. Mechanistically, research has elucidated specific pathways of communication, primarily through microbial metabolites such as short-chain fatty acids (SCFAs), secondary bile acids, and lipopolysaccharide (LPS), which can regulate host immunity, influence hormone conversion enzymes (deiodinases), affect intestinal barrier integrity, and alter the absorption of essential nutrients like iodine and selenium (36, 37). Thus, the gut-thyroid axis is not a metaphorical concept but a concrete physiological pathway, where the intestinal microbiome acts as a key interface between environmental factors and systemic endocrine-immune homeostasis, ultimately determining thyroid health trajectory.

## 2 Core mechanisms

### 2.1 Immune system regulation and autoimmunity initiation

The emerging “gut-thyroid axis” reveals that the intestinal microbiota and its metabolites play a foundational role in training the immune system (29, 30). When this complex ecosystem becomes imbalanced, it can disrupt immune tolerance and actively initiate the autoimmune processes underlying GD and HT (33–35). The mechanisms are multifaceted, involving direct microbial signaling, the immunomodulatory power of microbial metabolites, and the critical breakdown of the intestinal barrier.

This dysbiosis exerts precise control over the immune system by disrupting the balance of T-cell populations, a cornerstone of autoimmune thyroid disease (AITD) pathology (38). A healthy, diverse microbiota promotes the differentiation of regulatory T cells (Tregs), which are essential for maintaining immune tolerance and preventing autoimmunity. Conversely, dysbiosis, often characterized by a reduced *Firmicutes/Bacteroidetes* ratio, skews this balance (39). It drives the expansion of pro-inflammatory T helper 17 (Th17) cells while suppressing Treg populations, creating a dominant Th17/Treg ratio that fuels chronic inflammation and provides the cellular milieu for autoimmune attack (40, 41). This T-cell imbalance is not merely associative; specific “pathobionts” can translocate from the gut to systemic sites like the spleen and lymph nodes, where they directly induce the differentiation of TH17 cells and trigger a cascade of autoimmune antibody production.

The immunomodulatory effects are powerfully mediated by bacterial metabolites. Beneficial short-chain fatty acids (SCFAs), such as butyrate produced by bacteria like *Roseburia*, are critical for maintaining the intestinal barrier and possess potent anti-inflammatory properties that support Treg function (42). Dysbiosis reduces SCFA production, weakening the gut lining and initiating a “leaky gut.” This allows bacterial components like lipopolysaccharide (LPS) to enter the bloodstream (43–45). LPS is a potent endotoxin that binds to Toll-like receptors (e.g., TLR4) on immune cells, activating inflammatory pathways such as PI3K-AKT-IKKα and NF-κB, which drive systemic inflammation and can directly impair immune regulation. Furthermore, the microbiota influences immune responses through antigen presentation. The phenomenon of “molecular mimicry” occurs when immune cells activated by microbial antigens erroneously cross-react with structurally similar human thyroid proteins (e.g., thyroid peroxidase, thyroglobulin), thereby launching a targeted autoimmune assault on the thyroid gland (46).

### 2.2 Regulating the metabolism of thyroid hormones

The gut microbiota and its metabolites crucially regulate thyroid hormone (TH) metabolism through a multi-layered bidirectional relationship known as the gut-thyroid axis (38, 47). This regulation occurs primarily through direct enzymatic activity, enterohepatic recycling, and profound influences on intestinal barrier integrity and nutrient absorption. For instance, specific gut bacteria can produce enzymes like β-glucuronidase, which is responsible for deconjugating THs (like T4 and T3) that are excreted in bile (48). This process allows hormones to be reabsorbed back into the bloodstream, a cycle called enterohepatic circulation, directly affecting systemic TH levels. A functional intestinal barrier, maintained by key microbial metabolites, is essential for this process and for the absorption of TH synthesis cofactors.

Microbial metabolites serve as central mediators of this process, with SCFAs like butyrate and propionate playing particularly significant roles (49, 50). These SCFAs are not only the primary fuel for colonocytes, helping to maintain intestinal barrier integrity and prevent “leaky gut,” but they also exert systemic immunomodulatory and metabolic effects. A 2025 study in Graves’ orbitopathy demonstrated that butyrate therapy normalized serum TH levels in a mouse model (51). Separately, a 2025 project description indicates that *Bacteroides fragilis* and its metabolite propionate have shown therapeutic promise, working synergistically with conventional drugs to treat Graves’ disease by modulating the gut-thyroid axis (52). Conversely, dysbiosis and a disrupted barrier can lead to the translocation of bacterial components like lipopolysaccharide (LPS), promoting systemic inflammation that may dysregulate hepatic TH metabolism and deiodinase activity.

Finally, the gut microbiota profoundly influences the bioavailability of essential trace elements required for TH synthesis and activation. The absorption of iodine, selenium, iron, and zinc is directly modulated by the composition and metabolic activity of gut bacteria (53–55). For example, studies show the small bowel microbiome, which is the primary site for nutrient absorption, is significantly altered in patients with hypothyroidism and Hashimoto’s thyroiditis (HT) (56, 57). This dysbiosis can impair the uptake of these micronutrients. Furthermore, THs themselves regulate gut motility and the microenvironment, creating a feedback loop that shapes microbial composition. This tightly coupled, bidirectional interaction underscores the gut microbiome’s role as a master regulator of TH homeostasis.

### 2.3 Maintaining the intestinal barrier and systemic inflammation

The integrity of the intestinal barrier and the control of systemic inflammation are two interconnected pillars through which the gut microbiota and its metabolites influence thyroid health. A healthy gut microbiota is fundamental for maintaining the physical and functional integrity of the intestinal barrier, a selective gatekeeper that separates the gut lumen from the systemic circulation (19, 58). Key microbial metabolites, especially short-chain fatty acids (SCFAs) produced from dietary fiber fermentation, serve as the primary energy source for colonocytes and are crucial for reinforcing the tight junction proteins that seal the gut lining. Conversely, a state of gut dysbiosis, characterized by an imbalance in microbial communities, leads to a deficiency in these beneficial SCFAs. This weakens the tight junctions, resulting in increased intestinal permeability, a condition often termed “leaky gut”. This breach allows the translocation of bacterial components such as lipopolysaccharide (LPS) into the bloodstream, triggering a cascade of systemic immune activation.

Dysbiosis not only impairs barrier function but also directly fuels chronic, low-grade systemic inflammation that is detrimental to thyroid homeostasis. Once LPS translocates across the compromised gut barrier, it acts as a potent endotoxin. It binds to pattern recognition receptors like Toll-like receptor 4 (TLR4) on immune cells, activating major inflammatory signaling pathways such as NF-κB and PI3K-AKT (59, 60). This activation drives the production of pro-inflammatory cytokines (e.g., IL-6, TNF-α), creating a pervasive inflammatory milieu. Critically, this systemic inflammation can disrupt the finely tuned hypothalamic-pituitary-thyroid axis and may directly impair thyroid cell function. Recent research highlights the small bowel, the most metabolically active intestinal region where thyroid hormone absorption and nutrient uptake occur, as a key site in this process (61). A study found that patients with Hashimoto’s thyroiditis (HT) have a prevalence of small intestinal bacterial overgrowth (SIBO) more than twice that of healthy controls. SIBO itself induces local inflammation and further weakens the gut barrier, creating a vicious cycle that perpetuates both gut dysfunction and thyroid pathology (62, 63).

Furthermore, the inflammatory state driven by gut dysbiosis profoundly disrupts local and systemic immune regulation, directly promoting thyroid autoimmunity. The inflamed environment and altered microbial signals skew the differentiation of immune cells in the gut-associated lymphoid tissue. There is a well-documented shift toward an increase in pro-inflammatory T helper 17 (Th17) cells and a decrease in anti-inflammatory regulatory T (Treg) cells. This imbalance in the Th17/Treg ratio is a critical immune checkpoint observed in patients with autoimmune thyroid diseases (AITDs) like Graves’ disease and HT (64, 65). The breach of the intestinal barrier also exposes the immune system to a wider array of microbial antigens. Some of these antigens share structural similarities with thyroid proteins like thyroid peroxidase and thyroglobulin (Tg) (66, 67). Through a mechanism known as molecular mimicry, the immune system, primed to attack these bacterial antigens, can mistakenly cross-react and launch an autoimmune attack against the thyroid gland itself. Therefore, gut dysbiosis initiates a sequence of events—barrier breakdown, systemic inflammation, and immune dysregulation—that collectively erode self-tolerance and can ignite the autoimmune processes central to many thyroid disorders.

### 2.4 Affecting the absorption of trace elements

The gut microbiota profoundly influences the absorption and bioavailability of essential trace elements, which are fundamental for thyroid hormone synthesis, regulation, and overall gland function. This bidirectional interaction is central to the diet-gut-thyroid axis (47, 68). Dysbiosis can significantly alter the intestinal environment in ways that directly compromise the host’s ability to acquire and utilize crucial micronutrients such as iodine, selenium, iron, zinc, and copper. For example, specific gut bacteria and their metabolic activity are integral to the intestinal absorption of iodine, a cornerstone of thyroid hormone production. Similarly, the availability of selenium and zinc—critical cofactors for the enzymes that activate thyroid hormones—is heavily modulated by microbial metabolism (27). A 2024 clinical study provided direct evidence for this link, finding that patients with thyroid nodules had significantly lower serum levels of selenium and zinc compared to healthy controls, a condition that was associated with a reduced abundance of beneficial Lactobacillus bacteria (69). This demonstrates that gut dysbiosis can manifest as a functional micronutrient deficiency, even with adequate dietary intake, thereby creating a physiological state that hinders optimal thyroid function.

The mechanisms by which gut microbiota mediate this influence are multifaceted. Primarily, a healthy microbiota helps maintain an optimal intestinal pH and produces metabolites like short-chain fatty acids (SCFAs) that can enhance the solubility and absorption of minerals such as iron. Certain beneficial bacterial strains can also perform biotransformation, converting dietary compounds into forms with higher bioavailability; for instance, *Lactobacillus fermentum* has been shown to facilitate iron absorption through ferric-reducing activity (70). Conversely, a state of dysbiosis often leads to intestinal inflammation and impaired barrier function (“leaky gut”). This compromised environment disrupts the normal absorptive processes and can lead to competitive or antagonistic interactions. Pathogenic bacteria may directly sequester trace elements for their own growth, effectively depriving the host. Furthermore, chronic inflammation can alter the expression of host transporters involved in nutrient uptake. The relationship is also reciprocal, as the levels of these trace elements can, in turn, shape the composition of the microbiota itself. Dietary iodine supplementation has been shown to alter gut flora composition in mice, and iron levels significantly influence the growth of specific bacterial groups, with iron deficiency often correlating with dysbiotic states (71). This creates a complex feedback loop where thyroid-related nutrient status and gut microbial ecology continuously influence each other.

Ultimately, dysregulation of this microbial-nutrient axis can contribute directly to thyroid pathophysiology. Deficiencies in key elements like iodine, selenium, iron, and zinc are well-established risk factors for thyroid dysfunction and are frequently observed in autoimmune thyroid diseases (AITDs) such as Hashimoto’s thyroiditis and Graves’ disease. For example, iron is required for the efficient utilization of iodine by the thyroid peroxidase enzyme, and iron deficiency is common in patients with hypothyroidism. Selenium deficiency impairs antioxidant defenses and the conversion of the thyroid hormone T4 to the more active T3. Therefore, when dysbiosis induces or exacerbates such micronutrient deficiencies, it creates a permissive environment for thyroid dysfunction. This can manifest as impaired hormone synthesis, increased oxidative stress on thyroid tissue, and a disrupted immune response, all of which may lower the threshold for the development or progression of thyroid disorders. This mechanistic insight positions the gut microbiota as a critical modulator of thyroid health through its role as a gatekeeper for essential nutritional cofactors.

## 3 The specific mechanisms of key gut microbiota metabolites

The gut microbiota produces a complex array of metabolites that serve as primary signaling molecules in the gut-thyroid axis. These compounds, including short-chain fatty acids (SCFAs), secondary bile acids, indoles, and lipopolysaccharides (LPS), directly and indirectly modulate thyroid physiology by influencing immune responses, hormone metabolism, and systemic inflammation. Their production and balance are critically dependent on a healthy, diverse microbial community, and dysbiosis can lead to metabolite profiles that promote thyroid dysfunction and autoimmune disease.

### 3.1 Short-chain fatty acids

The primary mechanism of action for SCFAs, including butyrate, propionate, and acetate, centers on their profound systemic immunomodulatory effects and their role in maintaining intestinal barrier integrity (**Table 1**). As metabolites of dietary fiber fermentation, SCFAs are absorbed into the circulation where they act as signaling molecules. They inhibit histone deacetylases (HDACs), leading to epigenetic modifications that promote the differentiation and function of anti-inflammatory regulatory T cells (Tregs) while suppressing pro-inflammatory pathways such as NF-κB (72, 73). Concurrently, SCFAs serve as the primary energy source for colonocytes, which is critical for reinforcing the tight junctions of the intestinal epithelium. A deficiency in SCFAs compromises this barrier, a condition often termed “leaky gut,” allowing the translocation of immunogenic substances.

**Table 1:** Impact of short-chain fatty acids (SCFAs) on thyroid diseases.

SCFA type	Primary mechanisms of action	Potential impact on thyroid function & diseases
Butyrate	Immune Regulation: Acts as a histone deacetylase (HDAC) inhibitor, promoting the differentiation of regulatory T cells (Tregs) and suppressing pro-inflammatory responses.	Likely protective in autoimmune thyroid diseases. Its anti-inflammatory properties may dampen autoimmune attack on the thyroid and counteract disease-associated gut dysbiosis.
	Gut Barrier Integrity: Serves as the primary energy source for colonic epithelial cells, strengthening tight junctions and reducing intestinal permeability ("leaky gut").	
	Receptor-Mediated Signaling: Binds to receptors like GPR43/41 to modulate immune cell function.	
Propionate	Systemic Metabolic Regulation: Involved in hepatic gluconeogenesis, cholesterol synthesis, and appetite regulation.	May indirectly influence thyroid homeostasis by modulating systemic metabolism and immune tone. Particularly relevant in the context of metabolic disorders
	Immunomodulation: Shares anti-inflammatory properties with butyrate, influencing immune cells via GPR41/43 receptors.	
Acetate	Systemic Energy Metabolism: The most abundant SCFA; enters circulation and influences lipogenesis and energy expenditure in peripheral tissues.	May act as a broad systemic metabolic signal, potentially influencing the hypothalamic-pituitary-thyroid (HPT) axis or peripheral thyroid hormone metabolism. Its effects are likely foundational and widespread.
	Receptor Signaling: Exerts effects through receptors such as GPR43.	

The related disease tendency strongly associates SCFA deficiency with Autoimmune Thyroid Diseases, particularly Hashimoto’s thyroiditis and Graves’ disease. Dysbiosis, characterized by a reduction in SCFA-producing bacteria like Faecalibacterium, disrupts the delicate balance between Tregs and pro-inflammatory T helper 17 (Th17) cells (74). This skews the immune system towards a pro-inflammatory state, lowering the threshold for autoimmunity and facilitating the production of autoantibodies against thyroid antigens such as thyroid peroxidase (TPO) and thyroglobulin (Tg). The resulting chronic inflammation and loss of immune tolerance are hallmarks of AITD pathogenesis.

### 3.2 Secondary bile acids

Secondary bile acids, such as deoxycholic acid (DCA) and lithocholic acid (LCA), function as potent endocrine signaling molecules. They are produced by gut bacteria through the modification of primary bile acids from the liver (75, 76). Their core mechanism involves activating specific nuclear receptors, most notably the Farnesoid X Receptor (FXR) and the G-protein-coupled bile acid receptor TGR5. Activation of FXR in the liver regulates cholesterol and glucose homeostasis, while TGR5 activation in various tissues stimulates energy expenditure (77, 78). Crucially, signaling through these receptors also exerts broad anti-inflammatory effects, influencing systemic metabolic and inflammatory tone, which indirectly modulates thyroid hormone sensitivity and metabolic function.

The disease tendency linked to altered secondary bile acid profiles is most evident in Graves’ disease (GD). Clinical studies consistently show that patients with GD have significantly altered serum levels of specific secondary bile acids. For instance, reductions in DCA and ursodeoxycholic acid (UDCA) have been observed. This deficiency may diminish the crucial anti-inflammatory signaling via FXR and TGR5, thereby contributing to the immune dysregulation that characterizes GD. Furthermore, this dysregulation of bile acid metabolism is thought to be linked to the profound metabolic disturbances—such as rapid weight loss and altered lipid profiles—commonly seen in hyperthyroid states.

### 3.3 Indoles & derivatives

Indoles, such as indole-3-acetic acid, are derived from bacterial metabolism of the essential amino acid tryptophan (79). Their primary mechanism of action is as ligands for the aryl hydrocarbon receptor (AhR), a transcription factor highly expressed in immune cells and the gut epithelium (80, 81). Activation of AhR by beneficial indole derivatives is critical for maintaining mucosal immunity. It strengthens the intestinal barrier, promotes the production of protective cytokines like interleukin-22 (IL-22), and supports the maintenance of intraepithelial lymphocytes and Treg cells (82, 83). This reinforces the gut’s first line of defense and fosters a localized anti-inflammatory environment. Notably, synthetic indole derivatives have also been designed as highly selective agonists for Thyroid Hormone Receptor Beta (TRβ), demonstrating a direct pathway to influence thyroid hormone signaling.

The related disease tendency involves the consequences of an imbalance in tryptophan metabolism. Dysbiosis can shift this pathway toward the production of less beneficial or harmful metabolites. Reduced signaling through the AhR pathway can impair mucosal immunity and barrier function, contributing to systemic inflammation. This inflammatory milieu is a key feature in the pathogenesis of autoimmune thyroiditis. The potential direct modulation of TRβ by indole-based compounds also points to a novel therapeutic avenue for managing thyroid hormone-related metabolic disorders, such as dyslipidemia, without adversely affecting the heart (84).

### 3.4 Lipopolysaccharides

Lipopolysaccharides are potent pro-inflammatory endotoxins that constitute the outer membrane of Gram-negative bacteria. Their core mechanism is centered on triggering innate immune activation. Under conditions of dysbiosis and increased intestinal permeability (“leaky gut”), LPS translocates into the systemic circulation. It then binds to the Toll-like receptor 4 (TLR4) complex on immune cells and other tissues (85, 86). This binding activates major inflammatory signaling pathways, including NF-κB and MAPK, leading to the massive production of pro-inflammatory cytokines such as TNF-α, IL-1β, and IL-6. This systemic inflammation can directly suppress the hypothalamic-pituitary-thyroid (HPT) axis, disrupt the expression of thyroid hormone transporters and deiodinases, and create an oxidative environment that damages tissues (87). Gut microbiota and their metabolites have been proposed to modulate deiodinase (DIO1, DIO2, DIO3) expression and thyroid hormone transporter (MCT8, MCT10, OATP1C1) function, yet the evidence remains fragmented and often contradictory. Preclinical studies show that butyrate upregulates Dio2 expression in rodent brown adipose tissue via histone deacetylase (HDAC) inhibition and GPR41/43 signaling (18, 88), while secondary bile acids like deoxycholic acid activate TGR5 to enhance DIO2-mediated thermogenesis (89). However, human data are strikingly inconsistent: one study reported positive correlations between fecal butyrate and serum T3/T4 ratios in healthy adults (90), whereas another found no such association in Hashimoto’s thyroiditis patients, possibly due to differences in dietary fiber, medication use, or disease duration (34). Similarly, gut-derived LPS suppresses DIO2 and induces DIO3 via TLR4-NF-κB signaling in animal models, explaining the “euthyroid sick syndrome” (91), yet human ex vivo adipose tissue studies reveal that LPS effects are highly dependent on baseline inflammatory status and sex, with some samples showing no response (92). Regarding thyroid hormone transporters, evidence is even more nascent and contentious: antibiotic-induced gut depletion in mice reduced hepatic Mct10 expression by 40% in one study (93), but another found no change using a different antibiotic regimen, highlighting the critical influence of antibiotic class, treatment duration, and baseline microbiota composition (94). Crucially, no human study has directly examined whether prebiotics, probiotics, or fecal microbiota transplantation alter MCT8/MCT10 expression in thyroid-relevant tissues, leaving the link between gut dysbiosis and tissue-specific thyroid hormone resistance purely speculative (95). Thus, while mechanistic pathways are plausible in controlled models, the field must move beyond descriptive associations toward standardized longitudinal human cohorts and interventional trials to resolve these conflicts and establish causality.

The disease tendency most strongly associated with elevated circulating LPS levels—a state known as metabolic endotoxemia—is Hashimoto’s thyroiditis (HT). LPS-driven chronic low-grade inflammation is a key contributor to HT pathogenesis (96, 97). The inflammatory environment not only disrupts normal thyroid function but also promotes the activation of self-reactive lymphocytes and the breakdown of immune tolerance. Furthermore, LPS can act as an immune adjuvant, potentially lowering the threshold for and amplifying the immune response against thyroid autoantigens, thereby directly fueling the autoimmune destructive process in HT.

## 4 Intestinal flora imbalance and thyroid diseases

### 4.1 Hashimoto’s thyroiditis

A cornerstone of Hashimoto’s Thyroiditis (HT) pathology is the breakdown of the intestinal epithelial barrier, commonly termed “leaky gut.” This state is profoundly influenced by gut dysbiosis, a term that describes a significant imbalance in the gut microbiota’s composition and function. Recent clinical studies have demonstrated that patients with HT exhibit a markedly higher prevalence of small intestinal bacterial overgrowth (SIBO)—more than twice that of healthy individuals—regardless of their thyroid hormone levels (98). This local microbial overgrowth, particularly involving Gram-negative bacteria, leads to a cascade of detrimental effects. These bacteria weaken the tight junctions between intestinal epithelial cells, and their outer membrane component, lipopolysaccharide (LPS), translocates across the compromised barrier into the systemic circulation. LPS is a potent endotoxin that binds to Toll-like receptor 4 (TLR4) on immune cells, activating key inflammatory pathways such as NF-κB and driving the production of pro-inflammatory cytokines like tumor necrosis factor-alpha (TNF-α) and interleukin-6 (IL-6) (96, 97). This creates a state of chronic, low-grade systemic inflammation that serves as a fundamental trigger for immune dysregulation.

The systemic inflammation driven by a permeable gut and LPS translocation directly disrupts the delicate balance of the host immune system, creating an environment ripe for autoimmunity. In HT, a characteristic and well-documented immunological shift is observed: a significant imbalance between pro-inflammatory T helper 17 (Th17) cells and anti-inflammatory regulatory T (Treg) cells. Gut dysbiosis is a primary driver of this imbalance. A healthy gut microbiota promotes the differentiation and function of Tregs through metabolites like short-chain fatty acids (SCFAs), thereby maintaining immune tolerance. However, in HT, the dysbiotic state often features a reduction in SCFA-producing bacteria. This deficiency, coupled with the inflammatory signals from LPS, promotes the expansion of Th17 cells while suppressing Treg populations. This elevated Th17/Treg ratio fosters a pro-inflammatory milieu that lowers the threshold for autoimmune reactions. Critically, this dysregulated immune state, originating from the gut, facilitates the activation of self-reactive T and B lymphocytes, leading to the production of autoantibodies against thyroid-specific antigens such as thyroid peroxidase (TPO) and thyroglobulin (Tg), which are the serological and pathological hallmarks of HT.

### 4.2 Graves’ disease

Individuals with Graves’ disease exhibit a significantly altered gut microbiome composition compared to healthy individuals. This dysbiosis is characterized by a marked reduction in overall microbial diversity, including a specific deficiency in short-chain fatty acid (SCFA)-producing bacteria (99). Notably, beneficial genera such as *Bacteroides*, *Dialister*, *Coprococcus*, and *Megamonas* are depleted in Graves’ disease patients (100, 101). Concurrently, there is a shift in the relative abundance of major bacterial phyla, typically with an increase in Bacteroidetes and a decrease in Firmicutes, leading to a disrupted Firmicutes/Bacteroidetes ratio (102). Some studies also report an increased abundance of specific bacteria, including Lactobacillus, Prevotella, Veillonella, and Erysipelotrichia (103). This dysfunctional microbial ecosystem results in a significant change in the gut’s metabolic output, characterized by lower levels of beneficial SCFAs like butyrate, propionate, and acetate, and higher levels of pro-inflammatory bacterial components like lipopolysaccharide (LPS) circulating in the bloodstream.

The altered gut microbiota directly drives the autoimmune pathology of Graves’ disease by disrupting systemic immune homeostasis. The deficiency in SCFAs impairs the anti-inflammatory and immune-tolerant signals they normally provide. Simultaneously, elevated LPS (endotoxemia) acts as a potent inflammatory trigger by binding to Toll-like receptor 4 (TLR4) (85, 86). This dual imbalance promotes a shift in critical immune cell populations: it reduces the number and function of regulatory T cells (Tregs) while favoring the expansion of pro-inflammatory T helper 17 (Th17) cells, creating a pathogenic Th17/Treg imbalance. Crucially, *in vitro* studies show that LPS directly causes a disturbance in B-cell distribution—reducing memory B cells and increasing naïve B cells—which is a key step in the loss of immune tolerance (104, 105). This cascade of dysregulation lowers the threshold for autoimmunity and facilitates the activation of autoreactive B cells, ultimately leading to the production of thyroid-stimulating hormone receptor autoantibodies that characterize and drive Graves’ disease.

### 4.3 Hypothyroidism

The intestinal flora imbalance in hypothyroidism is characterized by a distinct and persistent disruption of the small bowel microbiome, which has profound physiological consequences for thyroid function (56, 106). Hypothyroidism itself is associated with specific bacterial patterns, such as a higher abundance of Neisseria in SIBO-negative patients (107). This dysbiosis leads to a decreased production of beneficial short-chain fatty acids (SCFAs) and an increase in serum lipopolysaccharide (LPS), a pro-inflammatory endotoxin.

This specific microbial imbalance disrupts the gut-thyroid axis through several key mechanisms. First, SIBO and the resulting inflammatory state weaken the intestinal barrier (“leaky gut”), allowing LPS and other bacterial products to enter the bloodstream. Second, the microbiome plays a crucial role in the absorption of thyroid hormone replacement (levothyroxine) and of critical micronutrients like iron, selenium, iodine, and zinc, which are essential cofactors for thyroid hormone synthesis (108–110). Third, the hypothyroid state itself may cause delayed gut motility, which could further promote bacterial overgrowth, creating a bidirectional, self-perpetuating cycle. The causal nature of this relationship is supported by animal studies, where fecal microbiota transplantation from hypothyroid patients to mice resulted in decreased thyroxine levels in the animals.

### 4.4 Thyroid nodules and thyroid cancer

The gut microbiome in individuals with thyroid nodules, particularly high-grade nodules, exhibits significant alterations in structure, function, and metabolic output. Research consistently demonstrates a reduced microbial diversity and a lower abundance of specific microbial genes in these patients compared to healthy controls and those with lower-grade nodules (69, 111, 112). This dysbiosis is not random; it involves a shift in the functional capacity of the gut microbiome, characterized by a significant deficiency in butyrate production pathways and an increased metabolic activity geared toward amino acid degradation. As butyrate is a key short-chain fatty acid (SCFA) with potent anti-inflammatory and immunomodulatory properties, its reduction represents a crucial functional disturbance. Intriguingly, this altered microbial metabolism appears to interface directly with the host’s endocrine regulation. For instance, the relative abundance of gut microbial pathways for L-histidine metabolism has been found to correlate with serum levels of thyrotropin-releasing hormone, a key regulator of the hypothalamic-pituitary-thyroid axis (113, 114). This suggests that gut dysbiosis may contribute to nodular pathology by both fostering a pro-inflammatory local environment and directly influencing the hormonal signaling that governs thyroid cell growth and function.

Advanced genetic epidemiological studies employing Mendelian Randomization analysis have begun to suggest a causal relationship between specific gut microbial taxa and the risk of developing thyroid nodules. This approach helps determine whether observed associations are likely to be causal. In one such study, certain microbial groups like *Desulfovibrionales*, *Prevotella_7*, and *Faecalibacterium* were identified as potential risk factors (115). Conversely, the *Lachnospiraceae* family was highlighted as having a potentially protective effect (69, 116). These findings indicate that an individual’s inherent gut microbiota composition may predispose them to thyroid nodule formation, reinforcing the concept of the gut-thyroid axis. Moreover, clinical studies have pinpointed specific, clinically relevant imbalances, such as a marked reduction in the beneficial bacterium Lactobacillus acidophilus in patients with both benign and malignant thyroid nodules compared to healthy individuals (117). This depletion is often paralleled by significantly lower serum levels of essential micronutrients like selenium and zinc, which are critical for thyroid hormone synthesis and antioxidant defense, further linking gut microbial health to thyroid nodule development through nutritional and metabolic pathways.

The link between microbial imbalance and thyroid cancer extends beyond the gut to a groundbreaking discovery: the existence of a distinct and dysbiotic intratumoral microbiome within thyroid cancer tissue itself. Research has confirmed that papillary thyroid carcinoma (PTC) tissues harbor a unique bacterial community that differs significantly from both benign thyroid nodules and adjacent normal tissue (118). This tumor microenvironment is characterized by reduced microbial diversity, and intriguingly, the diversity decreases further with increasing tumor size.

The intratumoral bacteria are not mere passive residents. They appear to be functionally engaged in the carcinogenic process. Integrative multi-omics analyses have revealed that the bacterial genera enriched in PTC tissues show positive correlations with the expression of key oncogenic signaling pathway genes, such as BRAF and KRAS (69, 118). This suggests that these tumor-resident microbes may actively contribute to cancer development and progression by interacting with and potentially modulating these critical cancer-driving pathways. This introduces the novel concept of the intratumoral microbiota as a local modulator of tumor biology. In parallel, studies on the gut microbiota in thyroid cancer patients also reveal significant dysbiosis, often characterized by a similar loss of diversity and beneficial taxa (119, 120). There is evidence of a reduced *Firmicutes*/*Bacteroidetes* ratio in the gut, a common marker of dysbiosis. This systemic gut imbalance may create a permissive, pro-inflammatory state that facilitates tumorigenesis, while the local intratumoral microbiome directly influences tumor cell signaling. This dual-level microbial involvement—systemic from the gut and local within the tumor—provides a compelling, multi-faceted mechanistic framework for how microbial communities influence thyroid cancer pathogenesis.

## 5 Clinical transformation and future prospects

The most direct path to clinical application involves microbiome-targeted interventions, including probiotics, prebiotics, and fecal microbiota transplantation (FMT). Preclinical and early clinical evidence suggests these approaches hold promise. For instance, supplementation with specific probiotic strains like *Lactiplantibacillus plantarum 299v* has shown potential in modulating the gut-thyroid axis and improving thyroid function. Recent clinical evidence has demonstrated that supplementation with *Lactiplantibacillus plantarum 299v* (Lp299v) for 12 weeks significantly enhances quality of life when combined with nutritional education, although it did not reduce anti-thyroid peroxidase antibody levels (121). In this study, the group receiving Lp299v alongside dietary guidance showed notable improvements in overall well-being, with benefits observed in 12 of 14 quality-of-life domains, compared to only 3 domains in the placebo group. Regarding dosage, research indicates that a minimum of 10^6^ CFU of Lp299v per day is required to achieve a therapeutic effect, with the commercially available preparation registered as safe for use at a recommended maximum of 2 capsules daily (121). More compellingly, a 2025 preclinical study demonstrated that butyrate supplementation not only normalized serum thyroid hormone and antibody levels in a Graves’ orbitopathy mouse model but also ameliorated orbital tissue pathology, showing direct therapeutic efficacy (122). FMT, a more comprehensive intervention, has emerged as a promising tool for recalibrating the gut ecosystem in diseases like Graves’ Disease, with effects that may extend via the gut-brain and gut-thyroid axes. However, translating these findings into standardized, effective clinical therapies faces substantial hurdles. Current evidence is often derived from small-scale studies, animal models, or preliminary clinical observations, and the efficacy, optimal dosing, and long-term safety of such interventions remain to be robustly validated in large-scale, longitudinal human trials. Moreover, the discovery of microbiome involvement in thyroid disease pathogenesis and treatment response opens the door to more precise management. Research is investigating how an individual’s unique microbial signature could be used to predict responses to standard therapies, like levothyroxine, or to identify individuals at higher risk for developing conditions like Small Intestinal Bacterial Overgrowth (SIBO), which is strongly linked to hypothyroidism.

The future of this field is anchored in the principle of precision medicine. The ultimate goal is to move beyond one-size-fits-all treatments to personalized management strategies based on a patient’s unique gut microbiota composition and metabolic profile. This involves developing reliable microbial biomarkers that can predict disease risk, stratify disease severity, and monitor therapeutic response, paving the way for truly tailored interventions. A key frontier is shifting the focus from treatment to primary prevention. A promising avenue is the proactive modulation of the gut microbiome through dietary and lifestyle interventions in at-risk populations. Given the strong bidirectional link, this also means that monitoring thyroid function in patients with gastrointestinal dysbiosis (and vice versa) may become a new screening strategy. Research must also expand beyond the bacterial component of the microbiome. Future investigations need to integrate the roles of the oral microbiome, virome (viruses), and mycobiome (fungi), which are critical but underexplored players in immune regulation and may significantly contribute to thyroid disease pathogenesis. Finally, establishing robust causality remains a paramount challenge. While advanced statistical methods like Mendelian Randomization have been employed in other contexts, more longitudinal studies and sophisticated model systems are needed to definitively determine whether gut dysbiosis is a driver or a consequence of thyroid disease, a distinction critical for developing effective treatments.

Current human studies on the gut microbiota–thyroid axis are constrained by small sample sizes that limit statistical power and generalizability, with most trials enrolling fewer than 100 participants. Furthermore, the near-absence of long-term prospective data makes it impossible to determine whether observed microbial alterations are causes or consequences of thyroid dysfunction. Additional limitations include significant heterogeneity in study designs, lack of standardized microbiota profiling methods, and insufficient adjustment for critical confounders such as diet, medication use, and iodine intake.

## 6 Conclusion

Based on the comprehensive evidence synthesized in this review, it is clear that the gut microbiota and its metabolites constitute a central, modifiable environmental factor in the pathogenesis and progression of thyroid diseases. Through the gut-thyroid axis, microbial metabolites such as short-chain fatty acids, secondary bile acids, and lipopolysaccharides directly regulate thyroid hormone metabolism, modulate systemic and local immune responses, maintain intestinal barrier integrity, and influence the absorption of essential nutrients, thereby influencing susceptibility to autoimmune disorders like Hashimoto’s thyroiditis and Graves’ disease, as well as functional impairments and neoplastic transformations. The bidirectional nature of this relationship, where thyroid status also shapes the gut ecosystem, underscores its complexity and therapeutic potential. Future research must prioritize establishing definitive causality in human populations through longitudinal and interventional studies, developing reliable microbial and metabolite-based biomarkers for precision diagnosis, and rigorously evaluating targeted interventions—from dietary modulation and specific probiotics to fecal microbiota transplantation. Ultimately, a deeper understanding of this intricate cross-talk promises to revolutionize the clinical management of thyroid diseases, shifting the paradigm from symptomatic treatment to a holistic, preventive, and personalized approach that addresses the root causes of dysregulation within the gut-thyroid axis.
